# Transcriptome sequencing reveals genome-wide variation in molecular evolutionary rate among ferns

**DOI:** 10.1186/s12864-016-3034-2

**Published:** 2016-08-30

**Authors:** Amanda L. Grusz, Carl J. Rothfels, Eric Schuettpelz

**Affiliations:** 1 Department of Botany, Smithsonian Institution, MRC 166 PO Box 37012, Washington, DC, 20013-7012 USA; 2 Department of Integrative Biology, University of California Berkeley, 1001 Valley Life Sciences Building, Berkeley, CA 94720-2466 USA; 3 Department of Biology, University of Minnesota Duluth, 1035 Kirby Drive, Duluth, MN 55812 USA

**Keywords:** *Adiantum*, Molecular evolution, Nucleotide substitution rate, Vittarioid ferns

## Abstract

**Background:**

Transcriptomics in non-model plant systems has recently reached a point where the examination of nuclear genome-wide patterns in understudied groups is an achievable reality. This progress is especially notable in evolutionary studies of ferns, for which molecular resources to date have been derived primarily from the plastid genome. Here, we utilize transcriptome data in the first genome-wide comparative study of molecular evolutionary rate in ferns. We focus on the ecologically diverse family Pteridaceae, which comprises about 10 % of fern diversity and includes the enigmatic vittarioid ferns—an epiphytic, tropical lineage known for dramatically reduced morphologies and radically elongated phylogenetic branch lengths. Using expressed sequence data for 2091 loci, we perform pairwise comparisons of molecular evolutionary rate among 12 species spanning the three largest clades in the family and ask whether previously documented heterogeneity in plastid substitution rates is reflected in their nuclear genomes. We then inquire whether variation in evolutionary rate is being shaped by genes belonging to specific functional categories and test for differential patterns of selection.

**Results:**

We find significant, genome-wide differences in evolutionary rate for vittarioid ferns relative to all other lineages within the Pteridaceae, but we recover few significant correlations between faster/slower vittarioid loci and known functional gene categories. We demonstrate that the faster rates characteristic of the vittarioid ferns are likely not driven by positive selection, nor are they unique to any particular type of nucleotide substitution.

**Conclusions:**

Our results reinforce recently reviewed mechanisms hypothesized to shape molecular evolutionary rates in vittarioid ferns and provide novel insight into substitution rate variation both within and among fern nuclear genomes.

**Electronic supplementary material:**

The online version of this article (doi:10.1186/s12864-016-3034-2) contains supplementary material, which is available to authorized users.

## Background

Improvements in technology and subsequent, rapidly diminishing costs have revolutionized molecular analyses of non-model lineages, such as ferns, and studies of these groups are finally moving into the “next generation” sequencing era [1–7]. Yet, even with the noted advances, researchers working on ferns have been somewhat limited in their ability to obtain and use genome-scale data, due primarily to the absence of an annotated genome sequence and difficulties posed by the large, highly repetitive genomes of most fern species [3, 7–9]. Fortunately, RNA sequencing of expressed genes can offer reasonable first insights into the evolution of fern genomes through the comparative lens of transcriptomics [8–19].

Among the many outstanding aspects of fern evolutionary biology that can be explored with transcriptome data are inconsistencies in molecular evolutionary rate across the fern tree of life [20–26]. Although especially pronounced in ferns, this phenomenon, of course, is not unique to the group. Unequal rates of sequence evolution are well documented throughout eukaryotes, both among lineages [27–34] and within individual genomes [35–38]. Many explanations for unequal rates of molecular evolution have been proposed, ranging from variation in generation time and fluctuations in mass-specific metabolic rate to disparities in latitude and environmentally available energy among lineages [27, 39–50]. But, the environmental and life history traits that are most commonly correlated with molecular evolutionary rate vary among branches of the eukaryotic phylogeny. In animals, evidence indicates that generation time or, more accurately, the rate of germline DNA replication [51] may be a strong driver of evolutionary rate [52, 53]. Other aspects, such as disparity in body size and the presence of damaging by-products of cellular metabolism, have also been implicated [40, 52].

Meanwhile, factors thought to influence molecular evolutionary rate in plants, particularly angiosperms, include environmental variables with potential mutagenic effects, like ultraviolet radiation and temperature [42, 44, 54, 55], as well as damaging metabolic byproducts and inefficient DNA repair [49]. Demographic effects involving population size, life history strategy (e.g., annual vs. perennial), habit (e.g., herbaceous vs. woody), and rates of mitosis have also been put forth to explain unequal evolutionary rates across plants [26, 33, 41, 56]. Although a consensus has not been reached regarding which of these factors most consistently influences substitution rates in plants, patterns of rate variation in many groups studied to date appear to be shared across cellular compartments (e.g., in bladderworts [32, 49] and *Veronica* [33]; but see [57]).

Within ferns, multiple lineages exhibit extreme heterogeneity in molecular evolutionary rate, most notably the filmy ferns [21], tree ferns [24], and vittarioid ferns [25]. But, in the studies to date, significant differences in lineage-specific evolutionary rate have been detected almost exclusively through comparisons of plastid gene regions. The one investigation to incorporate non-plastid loci examined just one gene from the nuclear genome and two from the mitochondrial genome [25], leaving significant shifts in evolutionary rates within and among the nuclear genomes of ferns effectively unexplored.

Here, we employ comparative transcriptomics to test whether previous observations of unequal molecular evolutionary rates among species at plastid loci are mirrored across the nuclear genome. We focus on the Pteridaceae, a cosmopolitan fern family comprising about 50 genera and more than 1000 species (roughly 10% of documented fern diversity) with ecological specializations to rupestral, aquatic, epiphytic, and terrestrial habitats [22, 23]. Taking a pairwise approach, we look for significant differences in rates of molecular evolution among the genomes of 12 species distributed broadly across the 3 largest clades in the family [22, 23] and evaluate life history characteristics potentially affecting these patterns. We focus particular attention on the *Adiantum* and vittarioid sister lineages, in an attempt to uncover genomic clues that may help to explain their disparate morphologies, ecologies, and life histories [25]. By examining 2091 nuclear gene regions, this study offers insight into the evolutionary drivers of divergence between *Adiantum* and the vittarioid ferns and sheds light on the mechanisms influencing evolutionary rates in the cosmopolitan fern family, Pteridaceae.

## Results

### Comparing relative substitution rates

**Fig. 1:**
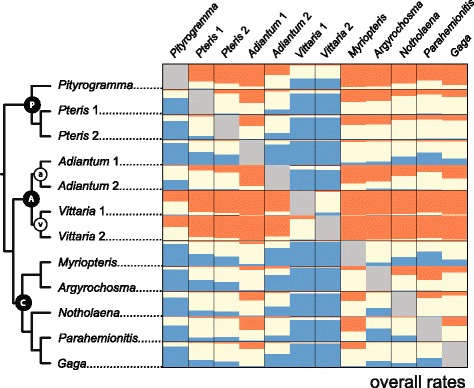
Summary of pairwise comparisons of overall substitution rates in the Pteridaceae for 2091 loci. Each box is colored based on the proportion of loci for which the row taxon is significantly slower (blue), faster (orange), or not significantly different (cream) than the corresponding column taxon. P = pteridoid clade, A = adiantoid clade, C = cheilanthoid clade, a = *Adiantum* clade, and v = vittarioid clade [22, 23]. For numerical values, see Additional file 2

**Fig. 2:**
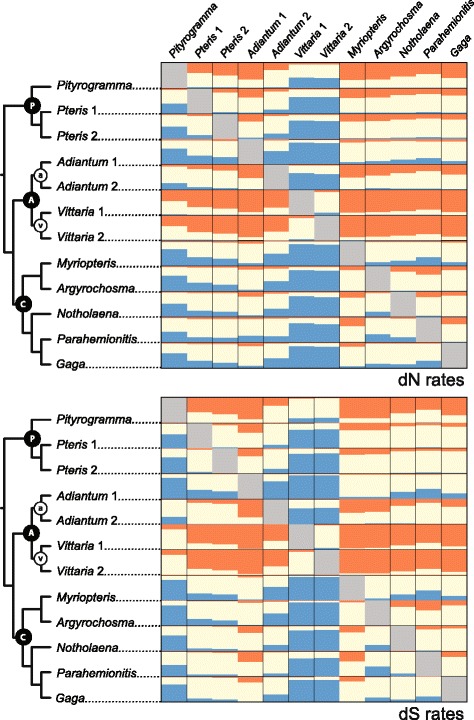
Summary of pairwise comparisons of nonsynonymous (dN) and synonymous (dS) substitution rates in the Pteridaceae for 2091 nuclear loci. Each box is colored based on the proportion for which the row taxon is significantly slower (blue), faster (orange), or not significantly different (cream) than the corresponding column taxon. P = pteridoid clade, A = adiantoid clade, C = cheilanthoid clade, a = *Adiantum* clade, and v = vittarioid clade [22, 23]. For numerical values, see Additional files 4 and 5

Relative rates of molecular evolution were examined at 2091 loci for 12 taxa from across the Pteridaceae (Additional file 1) [23]. We found a multitude of significant differences in overall molecular substitution rate among sampled taxa (Fig. 1; Additional file 2). For most (but not all) loci examined, the vittarioid ferns (v) consistently had the fastest relative substitution rates (Fig. 1). Representatives of the pteridoid clade (P, Fig. 1), *Pityrogramma* in particular, also showed fast substitution rates relative to other taxa (excluding the vittarioid ferns). The xeric-adapted cheilanthoid ferns (C), especially *Myriopteris*, exhibited consistently lower rates than other members of the Pteridaceae sampled. Separate pairwise comparisons of rate for individual substitution types (Additional file 3), nonsynonymous substitution rates (dN, Fig. 2; Additional file 4), and synonymous substitution rates (dS, Fig. 2; Additional file 5) each presented patterns of rate variation that were consistent with our pairwise comparisons of overall substitution rates (Fig. 1; Additional file 2).

### GO enrichment analyses

To detect whether any gene families contribute disproportionately to substitution rate variation for vittarioid ferns, we conducted gene ontology (GO) annotations (Additional files 6 and 7) and GO enrichment analyses. Specifically, we tested whether any particular GO categories were over- or underrepresented among the loci for which vittarioids had significantly faster or slower substitution rates. Because there is no annotated fern genome available, many orthogroups in our study did not map to a known GO category. For this reason, we carried out two separate tests of enrichment/purification for the faster loci and two for the slower loci: one that included all significantly faster or slower orthogroups, regardless of annotation status, and a second that included only those significantly faster or slower loci with known GO annotations. In our analyses blind to annotation status, we used a reference set (RS) including all 2091 loci (RS1; Additional file 8); in our analyses considering only annotated genes, we used a RS including only the subset of total loci with known annotations (RS2; Additional file 8). Thus we conducted four separate analyses, one for each test set (TS): all significantly faster loci (TS1; Additional file 8) versus RS1; significantly faster loci with annotations (TS2; Additional file 8) versus RS2; all significantly slower loci (TS3) versus RS1; and significantly slower loci with annotations (TS4) versus RS2 (see Additional file 8). When we restricted comparisons to those with GO annotations (TS2 vs. RS2 and TS4 vs. RS2), no GO categories showed evidence of being significantly enriched or purified among faster/slower vittarioid loci. However, when all loci were included in the reference set, whether annotated or not (RS1), one GO category was significantly purified among those annotated loci for which vittarioid ferns were significantly slower than other taxa (TS3): *GO:0050896: response to stimulus: physiological response to stimulus [exact]*.

### Tests for selection

**Fig. 3:**
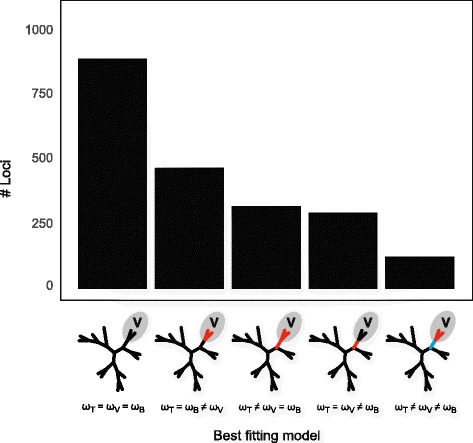
Number of loci for which a given dN/dS model best explains the data (*p* < 0.05). ω_V_ = estimated average dN/dS for vittarioid ferns; ω_B_ = estimated dN/dS for the branch subtending vittarioids; ω_T_ = estimated dN/dS for all taxa and branches excluding vittarioids and the subtending branch. For numerical values, see Table 1

In an effort to distinguish whether selective forces could be driving the fast substitution rates observed for vittarioid ferns, we also tested for significant differences in the ratio of nonsynonymous to synonymous substitutions (i.e., dN/dS or ω) for each of the 2091 orthogroups in our study. We used model comparisons to determine if dN/dS for the vittarioid crown group (ω_V_) and/or its stem branch (ω_B_) was statistically distinct from the rest of the phylogeny (ω_T_). For a plurality of loci (42.4 %), the best fitting model applied a single (shared) dN/dS across the three partitions (ω_T_ = ω_V_ = ω_B_; Fig. 3; Table 1), with vittarioids apparently not experiencing unique selective pressures. For an additional 22.3 % of loci analyzed, a two-rate model allowing ω_V_ to vary relative to the rest of the phylogeny (ω_T_ = ω_B_ ≠ ω_V_; Fig. 3; Table 1) best explained the data. Rates for roughly equal proportions of orthogroups (15.2 and 14.0 %; Fig. 3; Table 1) were best explained by a two-rate model with either a unique dN/dS for the vittarioid stem branch (ω_T_ = ω_V_ ≠ ω_B_) or the remainder of the tree (ω_T_ ≠ ω_V_ = ω_B_), respectively. The three-rate model (ω_T_ ≠ ω_V_ ≠ ω_B_) best explained the data for the fewest orthogroups (5.9 %; Fig. 3; Table 1).

**Table 1:** Best-fitting models of dN/dS (ω) across 2091 loci analyzed. For each model, the absolute number (and proportion) of loci for which that model best explained the data (according to AIC) is provided^a^

*Model*	*# Loci*	*Positive selection* *ω > 1*	*Neutral* *ω = 1*	*Purifying selection* *ω < 1*
ω_T_ = ω_V_ = ω_B_	888 (42.4 %)	1 (0.11 %)	0	887 (99.9 %)
ω_T_ = ω_B_ ≠ ω_V_	467 (22.3 %)			
ω_T_ = ω_B_		0	0	467 (100 %)
ω_V_		3 (0.64 %)	49 (10.5 %)	415 (88.9 %)
ω_T_ ≠ ω_V_ = ω_B_	318 (15.2 %)			
ω_T_		0	0	318 (100 %)
ω_V_ = ω_B_		0	2 (0.62 %)	316 (99.4 %)
ω_T_ = ω_V_ ≠ ω_B_	294 (14.0 %)			
ω_T_ = ω_V_		0	0	294 (100 %)
ω_B_		1 (0.34 %)	2 (0.68 %)	291 (98.9 %)
ω_T_ ≠ ω_V_ ≠ ω_B_	124 (5.9 %)			
ω_T_		0	0	124 (100 %)
ω_V_		0	8 (6.45 %)	116 (93.5 %)
ω_B_		1 (0.81 %)	1 (0.81 %)	122 (98.4 %)

^a^ω_T_ = estimated average dN/dS across all sampled taxa with the exclusion of vittarioid ferns and their subtending branch; ω_V_ = estimated average dN/dS for vittarioid ferns; ω_B_ = estimated dN/dS for branch subtending vittarioid ferns (see Fig. 3)

Regardless of which dN/dS model best explained the data for a given orthogroup and its corresponding partitions, the vast majority of regions analyzed had estimated ω values that were significantly less than 1 across all partitions, indicating that purifying selection has most likely been acting (Table 1). Neutrality was identified as the second most common evolutionary regime, with dN/dS estimates for ca. 3 % of loci failing to reject neutrality for at least one partition (i.e., with ω values not significantly different from 1; Table 1). Evidence for positive selection (i.e., an ω significantly greater than 1) was detected in only a handful (5; <1 %) of the loci surveyed (Table 1). Among the loci for which evidence of positive selection was detected, most had no known function based on existing GO annotations. Only a single locus determined to be under positive selection (ω_B_ partition, model: ω_T_ ≠ ω_V_ ≠ ω_B_; Table 1) mapped to a known GO category: *GO:0009987: cellular process: cell growth and/or maintenance [narrow]; cell physiology [exact]; cellular physiological process [exact].*

## Discussion

Here, we examine variation in molecular evolutionary rate among taxa spanning the three largest clades within the ecologically diverse fern family Pteridaceae [23]. Previous studies based primarily on plastid data uncovered considerable rate heterogeneity within this family, especially with respect to the morphologically simplified, epiphytic vittarioid lineage [23, 25]. However, it was unclear whether such patterns extended to the nuclear genomes in these ferns [58]. Using pairwise rate comparisons for 2091 nuclear loci, we ask whether the significant variation in molecular evolutionary rates previously reported for plastid loci is mirrored in fern nuclear genomes. Given the extremely fast plastid gene substitution rates that characterize vittarioid ferns, we focus particular attention on the nuclear gene substitution rates for members of this lineage.

Our results show that the variation in molecular evolutionary rate previously detected among members of the Pteridaceae (vittarioid ferns in particular) at plastid loci is reflected in their nuclear genomes (Figs. 1 and 2). Furthermore, we find that the vittarioid ferns are not alone in having significantly faster substitution rates (relative to other members of the family). Pteridoid ferns (*Pityrogramma*, especially) and at least one member of the *Adiantum* clade (*Adiantum* 2) are also characterized by relatively fast substitution rates (Figs. 1 and 2). Results from our model comparisons (ω) also indicate that unique selective pressures are probably not driving a rate increase at a plurality of loci in vittarioid ferns (Fig. 3).

While variation in molecular evolutionary rate has been documented across the tree of life, factors influencing substitution rates (e.g., body size, reproductive strategies, and life history) differ among lineages [40, 41, 48].

### Metabolism and evolutionary rates

Mutations are the fundamental fuel for molecular evolution, but the efficiency of DNA repair and the rate of fixation ultimately drive rates of molecular substitution across organismal genomes [35, 59]. Furthermore, many aspects of an organism’s biology that are critical to its survival can play an important role in generating mutations. One prominent example is the production of reactive oxygen species as a byproduct of cellular metabolism [40]. Because mitochondria are the sites of metabolism, they are expected to be a prime location for oxidative DNA damage [52, 60]. Given this, if the production of reactive oxygen species via metabolism is a major factor influencing substitution rates, we should expect increased substitution rates, especially with respect to CC → TT mutations, in mitochondrial genes [48, 61]. Some studies have found a strong signal for metabolic effects, or correlates thereof [62, 63], on mitochondrial substitution rates (but see [53, 64]), but doubt remains as to whether such effects extend to the nuclear genome [65–67].

Contrary to expectations based on the mitochondrial hypothesis of elevated evolutionary rates, Rothfels and Schuettpelz [25] found that increased substitution rates observed in plastid genes of vittarioid ferns were mostly not reflected in the two mitochondrial genes examined. Complementing this finding, our own comparisons of rate variation for individual nucleotide substitution types across nuclear genes in vittarioid ferns (and the Pteridaceae as a whole) do not show an overabundance of CC → TT mutations (Additional file 3). These results, in combination, indicate that mutagenic metabolic byproducts are probably not driving increased evolutionary rates in our focal group.

Among the most important outcomes of our study is the observation that patterns of rate heterogeneity among adiantoid (A) plastid genomes [25] are also detected in nuclear genome comparisons (Figs. 1 and 2, Additional file 3). Such parallel rate variation across cellular compartments is in conflict with the notion of DNA polymerase fidelity being a primary driver of molecular evolutionary rates in this group, owing to the fact that different DNA polymerases function in nuclei and plastids [41]. Therefore, causes of molecular evolutionary rate heterogeneity that result in asymmetrical differences among cellular compartments can be reasonably disregarded.

### Influence of generation time and DNA replication on rates

Like metabolism, DNA transcription, replication, and repair are essential yet potentially mutagenic biological processes [48, 68, 69]. Because animals retain a sequestered germ line, DNA replication has been frequently invoked to explain the strong correlation between their generation times and evolutionary rates [52, 53]. Plants, however, lack an early-sequestered germ line, so rates of mitotic division in the apical meristem are considered a better proxy for DNA replication frequency than actual time to reproduction [56]. Unfortunately, estimates for rates of mitosis in plants are lacking and the situation is further complicated in ferns, with time to reproduction also encompassing the period prior to fertilization when these plants exist in their independent haploid (gametophyte) life phase.

Some authors have hypothesized that ferns with an especially prolonged gametophyte phase may have an increased probability of fixation of somatic mutations in the progeny of meristematic cells [70, 71]. While estimates of overall generation time or rate of mitotic division in ferns are lacking, some estimates exist for the relative duration of the gametophyte life history stage [72]. Interestingly, vittarioid ferns, which we find to have the highest substitution rates within the Pteridaceae, are known for their long-lived, vegetatively reproducing gametophytes [72]. It is reasonable, then, to further consider the roles played by longevity and/or mitotic frequency in guiding evolutionary rates within ferns.

### Genome duplication and evolutionary rates

Not surprisingly, many facets of genome biology correlate with evolutionary rates, such as levels of gene expression (negatively correlated [73]), degree of promoter region methylation (positively correlated [74]), and extent of gene duplication (positively correlated [38, 75]). Additionally, genome structure itself (e.g., the position of plastid inverted repeats) has recently been shown to influence molecular substitution rates [76]. Our data indicate that genome duplication, in particular, may be an important characteristic influencing evolutionary rates in ferns. When viewed in light of published chromosome counts [77], our results reveal that taxa with the highest relative substitution rates within the Pteridaceae—*Vittaria*, *Pityrogramma*, *Pteris*, and *Adiantum* 2—tend to exhibit high ploidy levels compared to most other species sampled.

### Environmental influences on substitution rate in ferns

In this study, sampled taxa are specialized to a variety of environmental conditions, from the xeric-adapted cheilanthoid ferns (*Argyrochosma*, *Gaga*, *Myriopteris*, *Notholaena*, *Parahemionitis*), to terrestrial/rupestral members of the *Adiantum* and pteridoid clades, and even epiphytic, tropical lineages comprising vittarioid ferns. Abiotic and/or environmental variables thought to affect mutation rates, as well as rates of subsequent fixation, are numerous. Among them, the influences of latitude [44] (but see [78]), habitat [79, 80], environmentally available energy [45], temperature [42–44], ultraviolet radiation [44, 81], and species interactions [54] have gained the most traction as possible explanations for evolutionary rate discrepancies. In particular, De Vries [82] speculated that water stress could decrease the rate of metabolic activity in plants via reduced respiration. For this reason, if metabolism were a significant source of DNA substitutions, we might expect that ferns growing in drought-prone habitats would have reduced substitution rates. Our results do reveal decreased evolutionary rates across the xeric-adapted cheilanthoid ferns sampled, in agreement with De Vries’ hypothesis. However, epiphytes (like the fast evolving vittarioids) are also known for their adaptations to drought, even in tropical environments [72]. This inconsistency, combined with a general lack of support for a metabolic explanation (as noted above) only reinforces the conclusion that oxidative damage is probably not a major factor contributing to rate variation in ferns.

Another important environmental engine potentially driving an increase in DNA mutations is the presence of ultraviolet radiation. It has been shown that such radiation can leave the same genomic signature as mutagenic metabolic byproducts (CC → TT mutations) [83]. But, as discussed above, our survey of rate variation across different nucleotide substitution types (Additional file 3) does not uncover a notable increase in CC → TT mutations (relative to other substitution types) in taxa with faster overall substitution rates.

## Conclusions

This study reveals genome-wide variation in molecular evolutionary rate across the fern family Pteridaceae. Our data indicate that damaging metabolic byproducts and environmental influences such as ultraviolet radiation are likely not driving evolutionary rate differences in this group. Other previously proposed mechanisms thought to result in rate heterogeneity may be more plausible. Among them, the roles played by life history traits (including the rate of mitosis and duration of the haploid gametophytic life stage) and/or gene/genome duplication are especially compelling. Expanded sampling and additional studies of cytology and population genetics in ferns (including estimates of effective population sizes), as well as the assembly and annotation of nuclear and mitochondrial genomes, could further refine our understanding of the timing (e.g., crown versus stem), causes, and consequences of molecular evolutionary rate shifts in this diverse lineage of vascular plants.

## Methods

### Taxon sampling and transcriptome sequencing

Thirteen taxa from the fern family Pteridaceae were sampled for our analyses. Twelve represented our focal ingroup: *Adiantum aleuticum* (Rupr.) C. A. Paris (= *Adiantum* 1), *Adiantum raddianum* C. Presl (= *Adiantum* 2), *Argyrochosma nivea* (Poir.) Windham, *Gaga arizonica* (Maxon) Fay W. Li & Windham, *Parahemionitis cordata* (Hook. & Grev.) Fraser-Jenk., *Myriopteris rufa* Fée, *Notholaena montieliae* Yatsk. & Arbeláez, *Pityrogramma trifoliata* (L.) R. M. Tryon, *Pteris ensiformis* Burm. f. (= *Pteris* 1), *Pteris vittata* L. (= *Pteris* 2), *Vittaria appalachiana* Farrar & Mickel (= *Vittaria* 1), and *Vittaria lineata* (L.) Sm. (= *Vittaria* 2). One additional taxon, *Cryptogramma acrostichoides* R. Br., was chosen as the outgroup. *Cryptogramma* has been shown based on rigorous phylogenetic studies to be sister to the remainder of Pteridaceae (including all 12 ingroup taxa), making it an appropriate outgroup for our analyses [22, 23]. These 13 taxa were selected based on the availability of transcriptome data shared by members of the 1KP consortium [84]. Collaborators of 1KP contributed leaf material from which RNA was extracted and sequenced by the consortium, using Illumina paired-end technology (Additional file 1) [5, 15, 85].

### Transcriptome assembly and assessment of orthology

Orthology assessment largely followed the phylogenetic approach described in Yang and Smith [86] (for a visual summary of our particular approach to orthology assessment, as described here, see Additional file 9). First, transcriptomes were assembled by the 1KP consortium using SOAPdenovo-trans [87] as described in Johnson et al. [5] and Rothfels et al. [7]. Assembled nucleotide sequences were then translated into peptides using TransDecoder, implemented within the Trinity package [88, 89]. Next, the program ProteinOrtho was used to identify putatively orthologous regions among the translated amino acid sequences [90]. Orthologous regions (hereafter referred to as “orthogroups”) containing at least one transcript per species for each of our 13 sampled taxa were identified and retained for further analyses (Additional file 10: http://dx.doi.org/10.5061/dryad.rg22j).

Amino acid sequences of the orthogroups identified by ProteinOrtho were aligned automatically using MUSCLE [91, 92]. Phylogenetic analyses of the resulting alignments were conducted in RAxML version 8 [93] using a JTT + Γ model of protein evolution (-m = PROTGAMMAJTT). Resulting gene trees were rooted using Newick Utilities (nw_reroot) [94] with *Cryptogramma* specified as the outgroup. Because of the potential for short read lengths to limit the power of likelihood reconstructions, we assessed trees using a test of topology in the program TreeFix version 1.1.10 (likelihood model = treefix.models.raxmlmodel.RAxMLModel0.2.4; reconciliation model = treefix.models.duplossmodel.DupLossModel1.0.1) [95]. TreeFix optimizes gene tree topologies by maximizing a known species tree topology, and assumes that any discordance between a gene tree and the presumed species tree results from gene duplication and/or gene loss. Using the output trees from TreeFix (with ≥13 tips), any subtrees that did not disagree with our species tree hypothesis were pruned (prune_paralogs_MO.py) [86] and retained. Gene trees were then compared to the established species tree hypothesis [22] to determine whether a single sequence for each of our thirteen taxa was still present using the program OptRoot (-d = rf) [96]. Trees with 13 taxa and a topology consistent with the expected species tree (rf = 0) were retained for subsequent analyses.

Protein alignments for all retained orthogroups were then reverse-translated to their corresponding in-frame nucleotide sequence alignments using the back_translate.pl script. To remove plastid sequences, orthogroups were compared to the two published plastid genomes from the Pteridaceae, *Myriopteris lindheimeri* (Hook.) J. Sm. [4] and *Adiantum capillus-veneris* L. [97]. BLAST databases were created for each of the plastid genomes and a fasta-formatted file containing a single nucleotide sequence (from *Adiantum* 2) for each orthologous region was used to query against each of the plastid genome databases. Any orthogroups with a significant BLAST hit to either (or both) of the plastid genome sequences were discarded prior to analysis (e-value threshold = 1e^-5^).

### Testing for rate heterogeneity and signatures of selection

We executed a series of pairwise relative rate comparisons among our 12 focal taxa (*Cryptogramma* was used as the outgroup for all pairwise comparisons) using a likelihood ratio test approach implemented in the program HyPhy (PairwiseRelativeRate.bf) [98]. We first tested for variation in overall substitution rate, using a GTR model of sequence evolution, by comparing the likelihood scores obtained using a single, shared substitution rate for both taxa to the likelihood score obtained when the substitution rate for each taxon was estimated separately. In these analyses, we estimated branch lengths independently, with all other model parameters shared (i.e., using the “global” option); rate parameters were inferred by maximum likelihood and observed nucleotide frequencies were used as equilibrium frequencies (“observed” option). We also performed separate pairwise relative rate tests constraining (or not) each transition (“t_ct”, “t_ag”) and transversion (“t_ac”, “t_at”, t_gt”, t_cg”) type. For these analyses, substitution rates were again estimated using a GTR model of sequence evolution; all parameters were estimated independently for each branch (i.e., using the “local” option) and equilibrium frequencies were based on the observed nucleotide frequencies (i.e., using the “observed” option).

To look for signatures of selection, we performed two additional sets of pairwise relative rate comparisons (PairwiseRelativeRate.bf) [98]. One set of analyses compared the likelihood value estimated when the rate of nonsynonymous substitution (dN; “nonSynRate”) was constrained to be equal between the two focal taxa to the likelihood estimate obtained when dN was allowed to vary. A second set of analyses was performed in which synonymous substitution rate (dS; “synRate”) was examined in the same manner. Both sets of analyses (dN and dS) were implemented using the MG4 model of codon evolution and the universal genetic code (“universal” option); all parameters were estimated independently for each branch (“local” option).

An additional set of analyses comparing alternative models of dN/dS (= ω) across the tree for each locus (SelectionLRT.bf) [98] were run to determine the degree to which selection may be driving rate differences in vittarioid ferns. For these analyses, the tree was partitioned into three components (each with an associated dN/dS): the clade of interest (i.e., vittarioid ferns; ω_V_), the branch subtending the clade of interest (ω_B_), and the remainder of the tree (ω_T_). Likelihood model comparisons (AIC) were used to determine which of the following null hypotheses best explained the data available for each locus: (1) ω_T_ = ω_V_ = ω_B_; (2) ω_T_ = ω_V_ ≠ ω_B_; (3) ω_T_ = ω_B_ ≠ ω_V_; (4) ω_T_ ≠ ω_V_ = ω_B_; (5) ω_T_ ≠ ω_V_ ≠ ω_B_.

### GO enrichment analyses

Blast2GO [99–102], implemented within the CLC Main Workbench version 7.6.2 (Qiagen, Aarhus, Denmark), was used to BLAST, map, and annotate gene ontology (GO) categories corresponding to each of our 2091 analyzed orthogroups [103–106]. Following annotation, we performed GO enrichment analyses using goatools (find_enrichment.py, --fdr option, pval = 0.05) to determine whether any GO categories were significantly over- or underrepresented (i.e., enriched or purified) among the loci for which substitution rates in vittarioid ferns, in particular, were found to be significantly faster or significantly slower than at least one other focal taxon (for test sets analyzed, see Additional file 8). Significance was assessed using a Bonferroni Correction to account for multiple comparisons. Because a closely related, annotated genome was not available, many of our 2091 loci did not map to a known GO category. For this reason, we performed two separate GO enrichment analyses for each vittarioid rate category (i.e., significantly faster/slower loci), for a total of four separate comparisons. In the first comparison, the test set (TS1) included all loci for which vittarioid ferns were significantly faster than at least one other sampled taxon, and the reference set (RS1) included the entire population of loci, regardless of their annotation status. In the second comparison, the test set (TS2) included only annotated loci for which vittarioid ferns were significantly faster than at least one other sampled taxon, and the reference set (RS2) included only the population of loci with known GO annotations. In the third comparison, the test set (TS3) included all loci for which vittarioid ferns were significantly slower than at least one other sampled taxon, and the reference set (RS1) included the entire population of loci, regardless of their annotation status. In the final comparison, the test set (TS4) included only annotated loci for which vittarioid ferns were significantly slower than at least one other sampled taxon, and the reference set (RS2) included only the population of loci with known GO annotations (for all test and reference sets, see Additional file 8).

## Additional files

Additional file 1: Taxon sampling. Taxon sampling and corresponding voucher information for samples included in our analyses, as provided by contributors to the 1KP Project [84]. (PDF 76 kb) Additional file 2: Pairwise overall rate comparisons. Comparisons of pairwise relative substitution rate for 2091 orthogroups across sampled taxa from the Pteridaceae. (PDF 89 kb) Additional file 3: Pairwise rate comparisons per substitution type. Summary of pairwise relative rate comparisons for individual substitution types across sampled taxa from the Pteridaceae for 2091 loci. (PDF 580 kb) Additional file 4: Pairwise nonsynonymous rate comparisons. Comparisons of pairwise relative nonsynonymous rate (dN) for 2091 orthogroups across the Pteridaceae. (PDF 90 kb) Additional file 5: Pairwise synonymous rate comparisons. Comparisons of pairwise relative synonymous rate (dS) for 2091 orthogroups across sampled taxa from the Pteridaceae. (PDF 90 kb) Additional file 6: Orthogroup summary statistics. Summary statistics for the 2091 orthogroups analyzed in this study. (PDF 288 kb) Additional file 7: Summary of annotated GO categories. GO annotation distribution for loci corresponding to molecular function, biological processes, and cellular compartments. (PDF 403 kb) Additional file 8: GO analyses data files. Zipped folder containing ‘test set’ and ‘reference set’ loci for all GO analyses performed herein, along with a corresponding readme document describing individual analyses. (ZIP 3916 kb) Additional file 9: Flowchart for determining orthologous regions. Flow chart illustrating steps taken to identify single copy orthologous regions across our 13 sampled taxa. (PDF 344 kb) Additional file 10: Orthogroup data file. Zipped folder containing fasta-formatted reads identified by ProteinOrtho, used for all downstream orthogroup determination and analysis, along with readme document and relevant project-specific scripts (also available online via Dryad: http://dx.doi.org/10.5061/dryad.rg22j). (ZIP 12021 kb)
