# Supplementary material for: Transcriptome sequencing reveals genome-wide variation in molecular evolutionary rate among ferns
Source: BMC Genomics. 2016 Aug 30;17(1):692. doi: 10.1186/s12864-016-3034-2 (PMC5006594; doi:10.1186/s12864-016-3034-2)
Supplement: Additional file 1: — Taxon sampling. Taxon sampling and corresponding voucher information for samples included in our analyses, as provided by contributors to the 1KP Project [84]. (PDF 76 kb) [file 12864_2016_3034_MOESM1_ESM.pdf]

Additional file 1

Taxon sampling and corresponding voucher information for samples included in our analyses.[a]

Taxon	*1KP ID*	*Voucher*	*Material source*
Adiantum aleuticum (Rupr.) C. A. Paris	WCLG	*Rothfels 4090* (DUKE)	C. Rothfels
Adiantum raddianum C. Presl	BMJR	*Deyholos 2012-17* (ALTA)	M. Deyholos
Argyrochosma nivea (Poir.) Windham	XDDT	*Reeb 26-V-02/12* (DUKE)	C. Rothfels
Cryptogramma acrostichoides R. Br.	WQML	*Rothfels 4060.2* (DUKE)	C. Rothfels
Gaga arizonica (Maxon) Fay W. Li & Windham	DCDT	*Li 1290* (DUKE)	C. Rothfels
Myriopteris rufa Fée	GSXD	*Rothfels 3903* (DUKE)	C. Rothfels
Notholaena montieliae Yatsk. & Arbeláez	YCKE	*Rothfels 4098* (DUKE)	C. Rothfels
Parahemionitis cordata (Hook. & Grev.) Fraser-Jenk.	ZXJO	*DeGironimo 214/94* (NY)	D. W. Stevenson
Pityrogramma trifoliata (L.) R. M. Tryon	UJTT	*Rothfels 4109* (DUKE)	C. Rothfels
Pteris ensiformis Burm. f.	FLTD	*Soltis & Miles 3001* (FLAS)	D. Soltis
Pteris vittata L.	POPJ	*Stewart & Burris sn*	N. Stewart
Vittaria appalanchiana Farrar & Mickel	NDUV	*Li 1568* (DUKE)	F.-W. Li
Vittaria lineata (L.) Sm.	SKYV	*Rothfels 4120* (DUKE)	C. Rothfels

[a] All material was provided by contributors to the 1KP Project [82].
