# Supplementary material for: Transcriptome sequencing reveals genome-wide variation in molecular evolutionary rate among ferns
Source: BMC Genomics. 2016 Aug 30;17(1):692. doi: 10.1186/s12864-016-3034-2 (PMC5006594; doi:10.1186/s12864-016-3034-2)
Supplement: Additional file 3: — Pairwise rate comparisons per substitution type. Summary of pairwise relative rate comparisons for individual substitution types across sampled taxa from the Pteridaceae for 2091 loci. (PDF 580 kb) [file 12864_2016_3034_MOESM3_ESM.pdf]

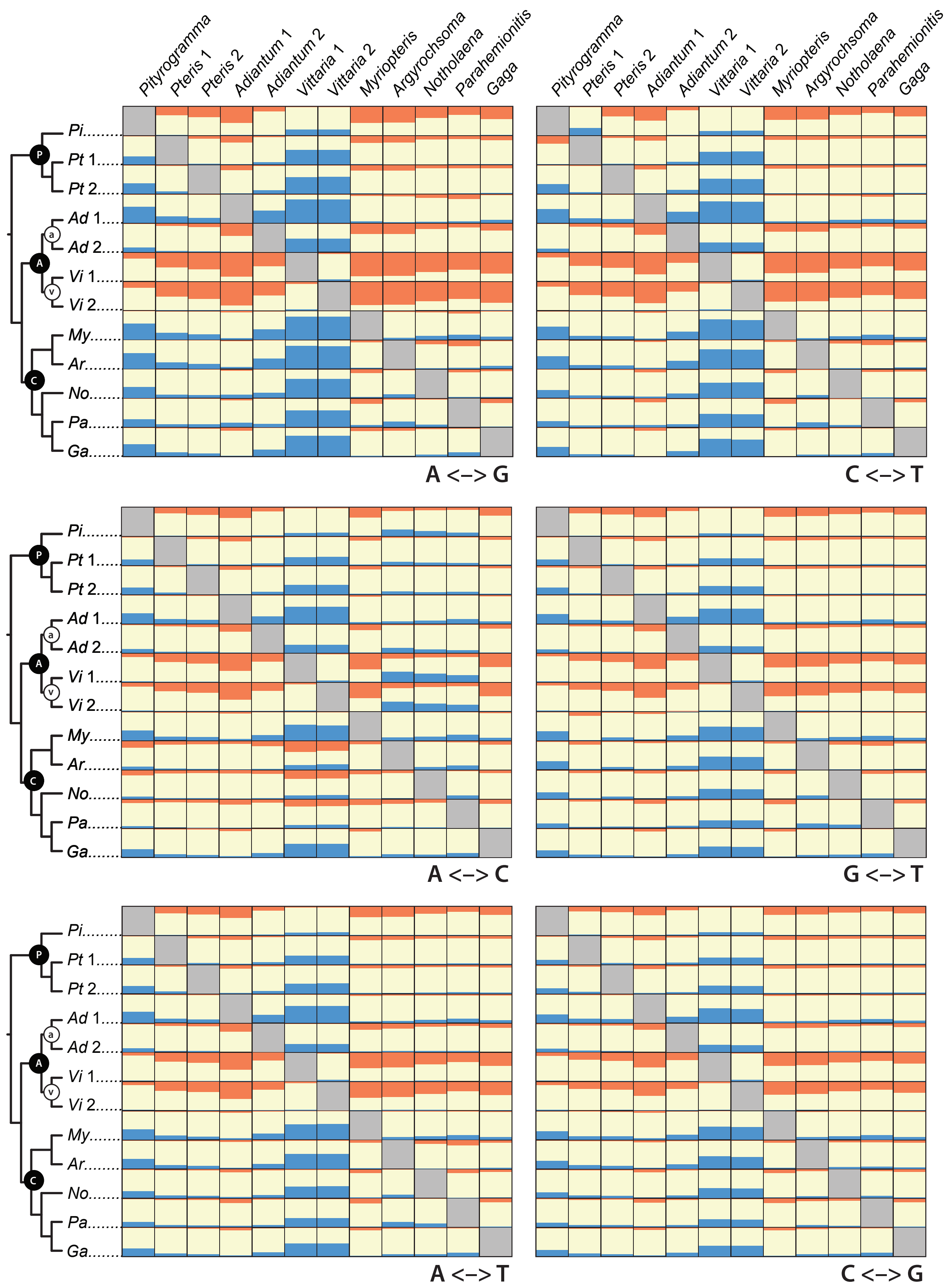

Additional file 3

Additional file 3

Summary of pairwise comparisons of substitution rates for particular substitution types across the Pteridaceae for 2091 loci. Each box is colored based on the proportion for which the row taxon is significantly slower (blue), faster (orange), or not significantly different (cream) than the corresponding column taxon. P = pteridoid clade, A = adiantoid clade, C = cheilanthoid clade, a = *Adiantum* clade, and v = vittarioid clade [22–23].
