# Supplementary material for: Transcriptome sequencing reveals genome-wide variation in molecular evolutionary rate among ferns
Source: BMC Genomics. 2016 Aug 30;17(1):692. doi: 10.1186/s12864-016-3034-2 (PMC5006594; doi:10.1186/s12864-016-3034-2)
Supplement: Additional file 5: — Pairwise synonymous rate comparisons. Comparisons of pairwise relative synonymous rate (dS) for 2091 orthogroups across sampled taxa from the Pteridaceae. (PDF 90 kb) [file 12864_2016_3034_MOESM5_ESM.pdf]

Additional file 5

Comparisons of pairwise relative synonymous substitution rate (dS) for 2091 orthogroups across the fern family Pteridaceae. [a]

	Pityrogramma	***Pteris 1***	***Pteris 2***	***Adiantum 1***	***Adiantum 2***	***Vittaria 1***	***Vittaria 2***	***Myriopteris***	***Argyrochosma***	***Notholaena***	***Parahemionitis***	***Gaga***
Pityrogramma												
**Fast**		57.2	66.5	85.6	30.9	1.3	1	83	83.4	62.9	47.5	69.5
**Insignificant**		42.1	33.1	14.2	68.6	80.2	80	16.9	16.5	37.1	51.9	30.2
**Slow**		0.6	0.4	0.2	0.5	18.5	19	0.1	0.2	0	0.5	0.3
Pteris 1												
**Fast**	0.6		7.6	36.7	1.8	0.6	0.5	28.3	28.2	11.2	7.4	13.8
**Insignificant**	42.1		89.8	62.7	85.2	25.4	24	71.1	70.9	88	90.5	84.6
**Slow**	57.2		2.7	0.6	13	74	75.5	0.6	1	0.8	2.1	1.6
Pteris 2												
**Fast**	0.4	2.7		30.7	1.2	0.3	0.3	23.5	22	9.2	8.1	10
**Insignificant**	33.1	89.8		68.5	80.8	20	18.8	75.8	77	88.7	88.2	87.7
**Slow**	66.5	7.6		0.9	17.9	79.7	80.9	0.7	1	2.2	3.7	2.3
Adiantum 1												
**Fast**	0.2	0.6	0.9		0.3	0.2	0.2	3.5	2	1.5	2.6	0.9
**Insignificant**	14.2	62.7	68.5		28.5	3.3	3.7	89.2	90.6	71.4	57.2	76.7
**Slow**	85.6	36.7	30.7		71.2	96.4	96.1	7.3	7.5	27.1	40.2	22.4
Adiantum 2												
**Fast**	0.5	13	17.9	71.2		0.2	0.3	55.1	50.6	25.2	12.5	30.6
**Insignificant**	68.6	85.2	80.8	28.5		34.4	33.8	44.8	46.2	72.6	86	68.8
**Slow**	30.9	1.8	1.2	0.3		65.3	65.9	0.1	3.2	2.2	1.4	0.6
Vittaria 1												
**Fast**	18.5	74	79.7	96.4	65.3		1.5	95.1	94.4	83.5	73.9	88.4
**Insignificant**	80.2	25.4	20	3.3	34.4		92.1	4.8	5.4	16.1	25.6	11.4
**Slow**	1.3	0.6	0.3	0.2	0.2		6.4	0.1	0.2	0.4	0.4	0.2
Vittaria 2												
**Fast**	19	75.5	80.9	96.1	65.9	6.4		95	94.4	85.6	75.1	88.1

**Insignificant**	80	24	18.8	3.7	33.8	92.1		4.9	5.4	14.2	24.6	11.7
**Slow**	1	0.5	0.3	0.2	0.3	1.5		0.1	0.2	0.2	0.3	0.2
Myriopteris												
**Fast**	0.1	0.6	0.7	7.3	0.1	0.1	0.1		0.4	0.4	0.8	0.9
**Insignificant**	16.9	71.1	75.8	89.2	44.8	4.8	4.9		93	75.5	58.7	81.1
**Slow**	83	28.3	23.5	3.5	55.1	95.1	95		6.6	24.1	40.6	18
Argyrochosma												
**Fast**	0.2	1	1	7.5	3.2	0.2	0.2	6.6		24.1	39	17.4
**Insignificant**	16.5	70.9	77	90.6	46.2	5.4	5.4	93		75.7	60.2	81.8
**Slow**	83.4	28.2	22	2	50.6	94.4	94.4	0.4		0.2	0.9	0.8
Notholaena												
**Fast**	0	0.8	2.2	27.1	2.2	0.4	0.2	24.1	0.2		12.2	8.3
**Insignificant**	37.1	88	88.7	71.4	72.6	16.1	14.2	75.5	75.7		86.9	91.4
**Slow**	62.9	11.2	9.2	1.5	25.2	83.5	85.6	0.4	24.1		0.9	0.3
Parahemionitis												
**Fast**	0.5	2.1	3.7	40.2	1.4	0.4	0.3	40.6	0.9	0.9		20.8
**Insignificant**	51.9	90.5	88.2	57.2	86	25.6	24.6	58.7	60.2	86.9		78.6
**Slow**	47.5	7.4	8.1	2.6	12.5	73.9	75.1	0.8	39	12.2		0.7
Gaga												
**Fast**	0.3	1.6	2.3	22.4	0.6	0.2	0.2	18	0.8	0.3	0.7	
**Insignificant**	30.2	84.6	87.7	76.7	68.8	11.4	11.7	81.1	81.8	91.4	78.6	
**Slow**	69.5	13.8	10	0.9	30.6	88.4	88.1	0.9	17.4	8.3	20.8	

[a] Rows labeled “Fast”, “Insignificant”, and “Slow” indicate the fraction of total loci for which the row taxon is significantly faster, not significantly different, or significantly slower than the corresponding column taxon, respectively. For a visualization of these proportions, see Fig. 2B.
