# Supplementary figures and images for: Transcriptome sequencing reveals genome-wide variation in molecular evolutionary rate among ferns
Source: BMC Genomics. 2016 Aug 30;17(1):692. doi: 10.1186/s12864-016-3034-2 (PMC5006594; doi:10.1186/s12864-016-3034-2)

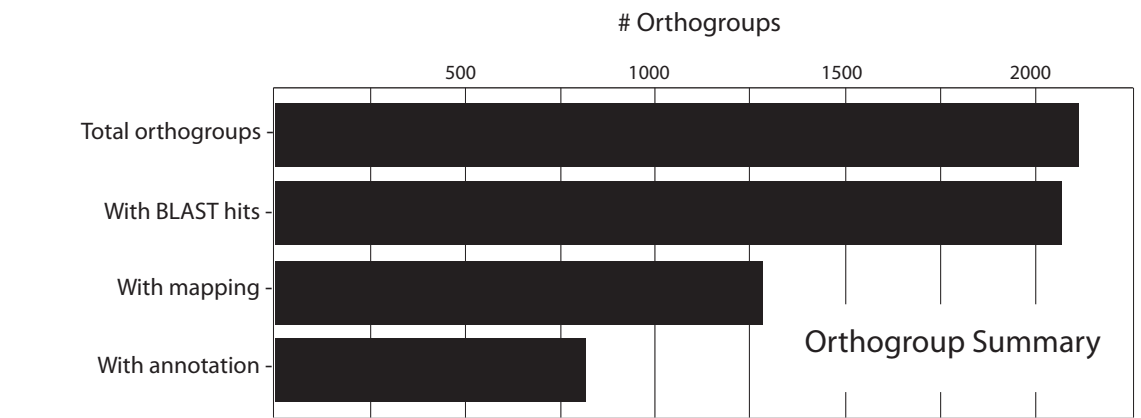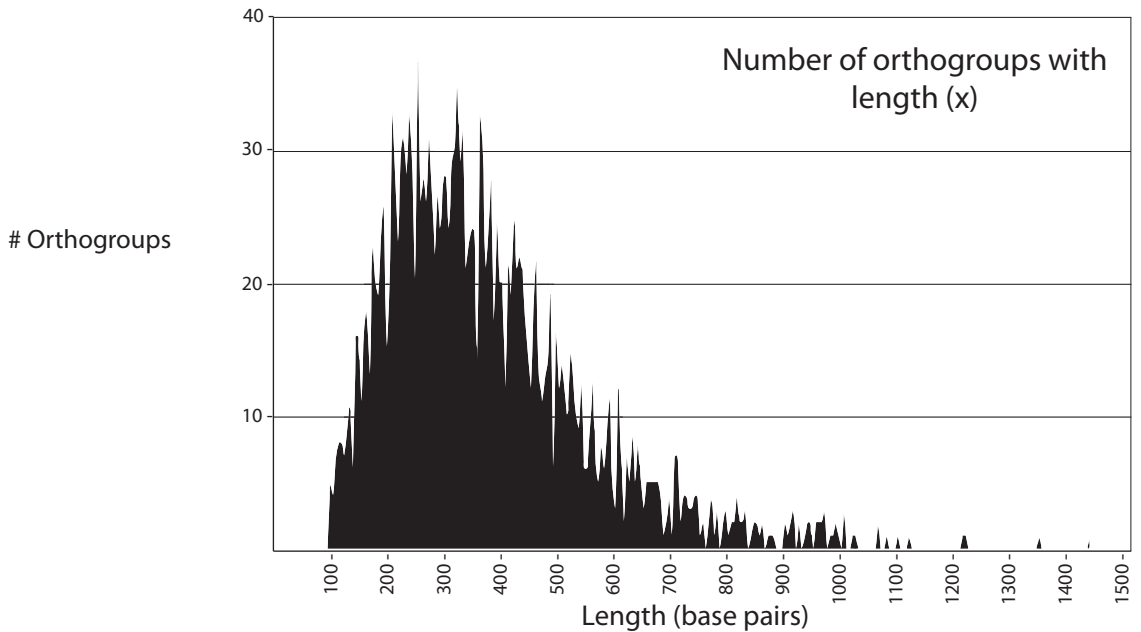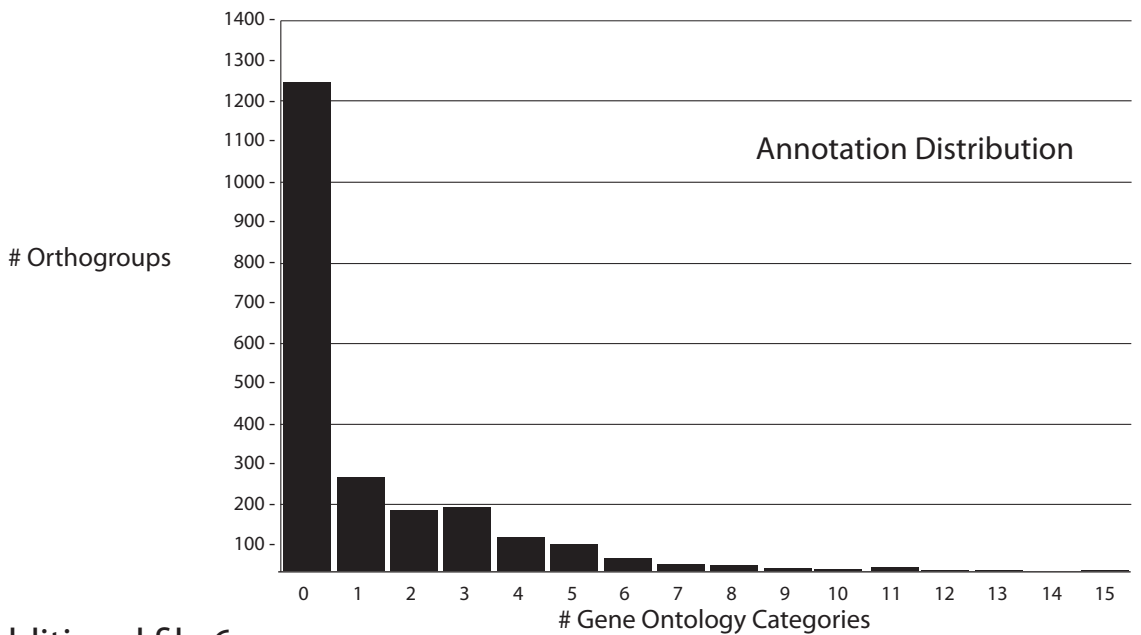

Supplement: Additional file 6: — Orthogroup summary statistics. Summary statistics for the 2091 orthogroups analyzed in this study. (PDF 288 kb) [file 12864_2016_3034_MOESM6_ESM.pdf]
