# Supplementary material for: Transcriptome sequencing reveals genome-wide variation in molecular evolutionary rate among ferns
Source: BMC Genomics. 2016 Aug 30;17(1):692. doi: 10.1186/s12864-016-3034-2 (PMC5006594; doi:10.1186/s12864-016-3034-2)
Supplement: Additional file 7: — Summary of annotated GO categories. GO annotation distribution for loci corresponding to molecular function, biological processes, and cellular compartments. (PDF 403 kb) [file 12864_2016_3034_MOESM7_ESM.pdf]

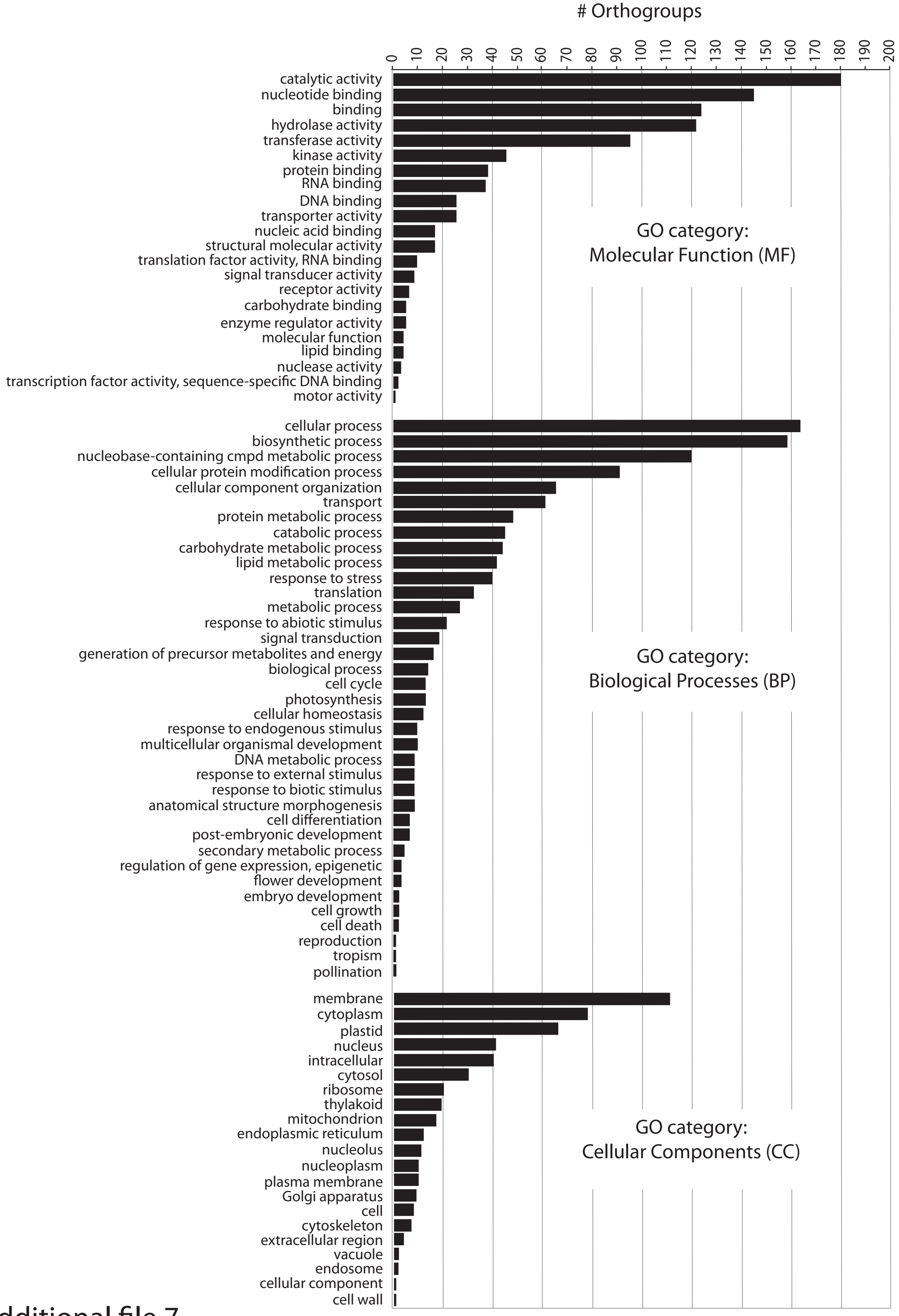

Additional file 7

Additional file 7

Summary of GO categories corresponding to molecular function, biological processes, and cellular compartments, and the number of orthogroups with annotations assigned to each.
