# Supplementary material for: Transcriptome sequencing reveals genome-wide variation in molecular evolutionary rate among ferns
Source: BMC Genomics. 2016 Aug 30;17(1):692. doi: 10.1186/s12864-016-3034-2 (PMC5006594; doi:10.1186/s12864-016-3034-2)
Supplement: Additional file 9: — Flowchart for determining orthologous regions. Flow chart illustrating steps taken to identify single copy orthologous regions across our 13 sampled taxa. (PDF 344 kb) [file 12864_2016_3034_MOESM9_ESM.pdf]

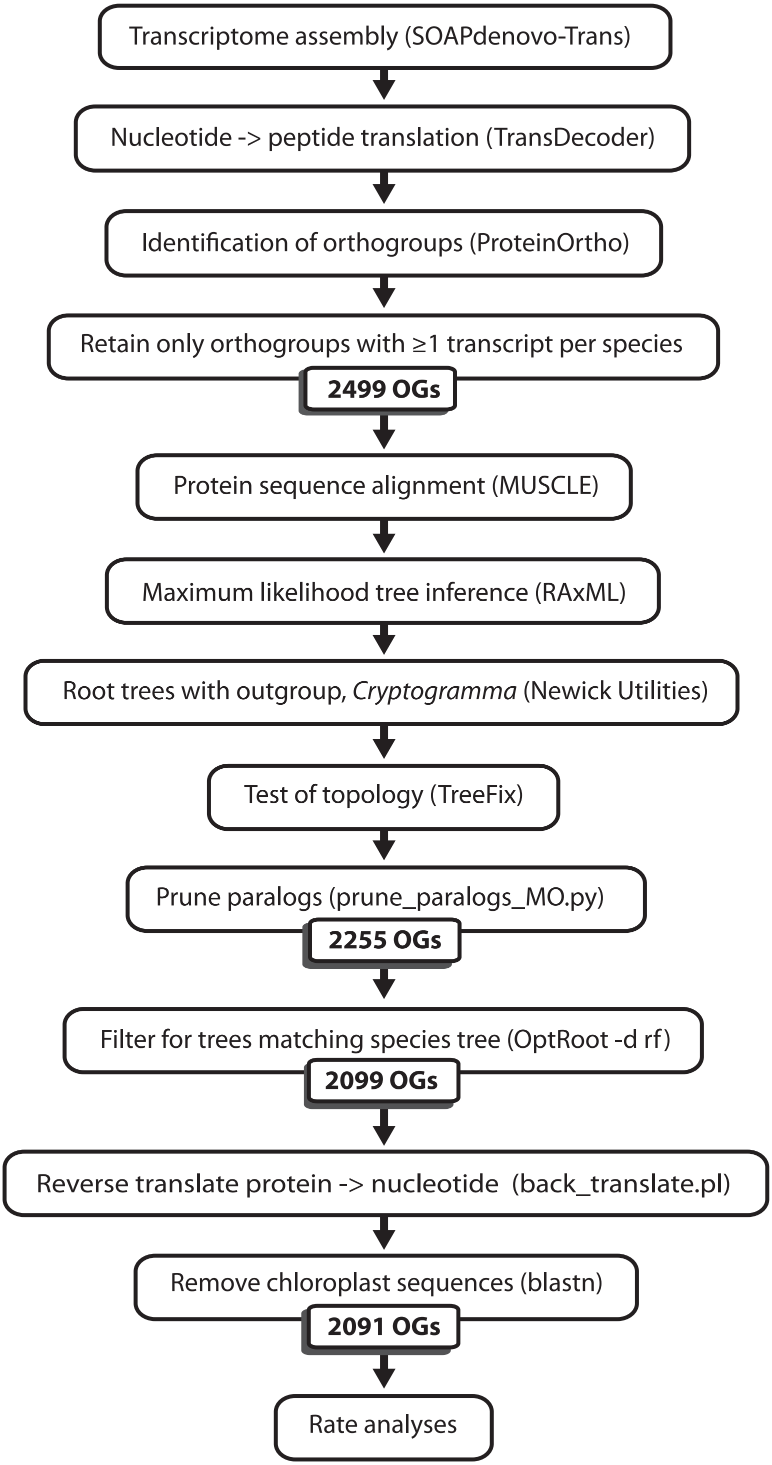


Additional file 9

**Additional file 9**

Flow chart illustrating steps taken toward identifying and analyzing single copy orthologous regions across our 13 sampled taxa.
